# Barcoding Fauna Bavarica: Myriapoda – a contribution to DNA sequence-based identifications of centipedes and millipedes (Chilopoda, Diplopoda)

**DOI:** 10.3897/zookeys.156.2176

**Published:** 2011-12-20

**Authors:** Jörg Spelda, Hans S. Reip, Ulla Oliveira–Biener, Roland R. Melzer

**Affiliations:** 1 Section Arthropoda Varia, Bavarian State Collection of Zoology, Münchhausenstraße 21, 81247 Munich, Germany; 2 Department of Soil Zoology, Senckenberg Museum of Natural History Görlitz, P.O. Box 300154, 02806 Görlitz, Germany

**Keywords:** Chilopoda, Diplopoda, COI barcoding, Bavaria, Germany

## Abstract

We give a first account of our ongoing barcoding activities on Bavarian myriapods in the framework of the Barcoding Fauna Bavarica project and IBOL, the International Barcode of Life. Having analyzed 126 taxa (including 122 species) belonging to all major German chilopod and diplopod lineages, often using four or more specimens each, at the moment our species stock includes 82% of the diplopods and 65% of the chilopods found in Bavaria, southern Germany. The partial COI sequences allow correct identification of more than 95% of the current set of Bavarian species. Moreover, most of the myriapod orders and families appear as distinct clades in neighbour-joining trees, although the phylogenetic relationships between them are not always depicted correctly. We give examples of (1) high interspecific sequence variability among closely related species; (2) low interspecific variability in some chordeumatidan genera, indicating that recent speciations cannot be resolved with certainty using COI DNA barcodes; (3) high intraspecific variation in some genera, suggesting the existence of cryptic lineages; and (4) the possible polyphyly of some taxa, i.e. the chordeumatidan genus *Ochogona*. This shows that, in addition to species identification, our data may be useful in various ways in the context of species delimitations, taxonomic revisions and analyses of ongoing speciation processes.

## Introduction

Molecular species identification based on sequence diversities in the Folmer segment of mitochondrial COI DNA has been under intense study for some years (Hebert et al. 2003, Savolainen et al. 2005). For the identification of a wide set of species, reference barcode libraries are needed; therefore, various projects are currently building such libraries by mass sequencing. The Barcoding Fauna Bavarica project (http://www.faunabavarica.de, Haszprunar 2009, Hausmann et al. 2011a,b), in close association with IBOL, the International Barcode of Life (http://ibol.org/), the DNA bank facility at Zoologische Staatssammlung München (ZSM) (http://www.zsm.mwn.de/dnabank/, Gemeinholzer et al. 2011), and the GLOMYRIS project of the Global Biodiversity Information Facility (GBIF) (http://www.gbif.de/evertebrata2/glomyris), aims to barcode all animal species in Bavaria, i.e. some 35000 species, representing 85% of the species found in Germany.

Among the Chilopoda and Diplopoda, the 146 species known from Bavaria cover 73% of the fauna of Germany. Hence, the first aim of our study is to establish a barcode reference library for Bavarian Myriapoda that will be expanded step by step (the dataset treated in this paper can be accessed in Barcode of Life Data Systems (BOLD; Ratnasingham and Hebert 2007, http://www.boldsystems.org) under ‘MYFBA (Fauna Bavarica Myriapoda public 1)’ as part of the campaign ‘Fauna Bavarica’). Moreover, myriapods found in our studied area cover many of the numerous species and subspecies of uncertain morphology-based species delimitations described in the huge works of K.W. Verhoeff (e.g. 1934, 1935) and C. Attems (e.g. 1927) many of which need taxonomic revision. Our work aims to provide morphology-independent sets of characters to enable us to check against the descriptions and species delimitations, and therefore to draw new conclusions about the validity of these species. This is also important since in the Bavarian Alps numerous species are found which are relicts of speciation processes that occurred during and after the last glaciation periods. Our barcodes will provide a basis or a test for these analyses (e.g. Pilz et al. 2008). Furthermore, barcoding of myriapods is of particular interest since in many species, i.e. in many diplopods such as the family Julidae, only a small fraction of the specimens (only adult males) can be identified using morphological sets of characters. Conversely, DNA barcoding allows the determination of all developmental stages from the egg to the male or female adults. In the future, DNA barcoding will therefore allow the identification of all life stages of these taxa instead of adult males only.

In the present paper we give an overview of our ongoing barcoding activities, which so far cover 73% of all Bavarian Chilopoda and Diplopoda. In addition to conventional analysis of the actual dataset based on our BOLD data, we give examples of how our barcodes will contribute to taxonomic revisions and to analyses of past and ongoing speciation processes.

## Material and methods

### Sampling

**Figure 1.**
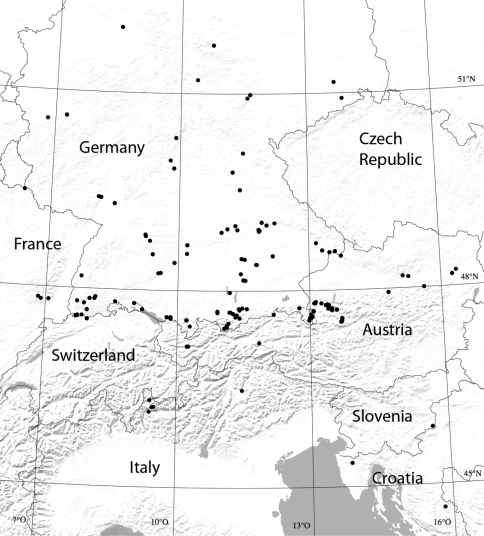
Map of sampled areas (dots). For checks of intraspecific variability of COI sequences, localities in Bavaria, but also elsewhere within the species’ areas of distribution, have been sampled and analyzed (sampling data from November 2008 to November 2010; a few specimens from northern Spain omitted).

To cover the variability within species, numerous samples from locations inside and outside Bavaria have been included. Besides the centipedes and millipedes known to occur in Bavaria, species that might be found there in the future have also been included, as well as close relatives of the known Bavarian species (Fig. 1). Since the study arose from the ‘Barcoding Fauna Bavarica’ project, sampling was restricted to a few individuals per species. We have tried to include material from all four major Bavarian faunal regions as defined in Voith (2004), provided that the species occurred in all of them. If this was not the case, additional sampling took place in adjacent countries. Attempts to amplify and sequence museum material (stored in denatured 75% ethanol), mainly from the more than 70 year-old Verhoeff collection housed at the ZSM, have failed. Hence we had to use newly sampled material less than two years old. This fresh tissue material was ideally stored in reagent-grade 96% ethanol which was exchanged several (3-4) times. In practice it was sometimes unavoidable to use material stored in about 75% ethanol for some days or months before replacement with 96% ethanol. All specimens used for sequencing have been photographed, as required by the Canadian Centre for DNA Barcoding (CCDB). Most of these photos were taken of live specimens in the field and are available online via BOLD. Taxonomy and nomenclature is based on the Bavarian list by Spelda (2006), except for a few updates reflecting more recent taxonomic decisions, e.g. taxa raised to species rank, new synonymies and new combinations. A website has been established for ‘Barcoding Fauna Bavarica’ (http://www.faunabavarica.de) that continuously updates project progress, such as lists of species and their barcode coverage.

### DNA sequencing

Sequencing was carried out at the CCDB, using the standard protocols of IBOL (http://www.dnabarcoding.ca/pa/ge/research/protocols). For reasons of performance, so far only the C_LepFolF and C_LepFolR primers have been used for PCR and sequencing. Barcoded voucher specimens are stored at ZSM, and DNA extracts from the specimens at the CCDB and the ZSM’s DNA bank facility (http://www.zsm.mwn.de/dnabank/). Specimen data, images and DNA sequences will be available on BOLD. BOLD numbers are given for each specimen in the depicted NJ trees (Figs. 8–11). These allow the tracking of our sequences in BOLD and GenBank, respectively.

Sequencing failed for about 30% of the species. Sometimes whole genera (*Trachysphaera*, *Ommatoiulus*, *Megaphyllum*, *Mycogona*), and sometimes species-level taxa (*Glomeris undulata* s.l., *Leptoiulus simplex*-group) were reluctant to barcoding. In these cases we either obtained no barcodes, or less than a quarter of the specimens were successfully barcoded. Hence, barcoding success of single samples was somewhat unpredictable. It seems that minor differences in tissue composition and protocol determine whether or not a sample runs; e.g., in one particular plate all *Megaphyllum* and *Ommatoiulus* were amplified successfully, whereas they had failed before.

### Data analysis

Resulting data for the myriapods treated here are taken from the respective tools included in BOLD, and calculated using the Kimura 2 Parameter (K2P) model. Sequences were imported into PAUP* (Swofford 2003) as Fasta files, and tree statistics were calculated using the bootstrap algorithm of PAUP* with 10 replicates and a neighbour-joining/UPGMA search. Only groups with a frequency above 50% were retained for consensus tree reconstruction.

## Results

### Data analysis

**Figure 2.**
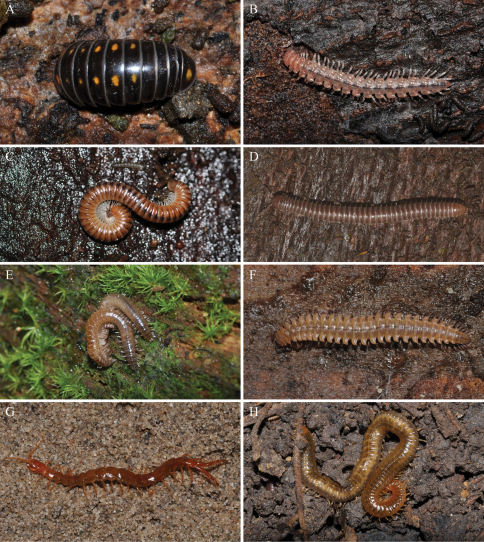
Some Bavarian myriapods for which barcodes are now available. **A.** *Glomeris pustulata* Latreille, 1804. **B.** *Polydesmus helveticus* Verhoeff, 1894. **C.** *Cylindroiulus boleti* (C. L. Koch, 1847). **D.** *Unciger foetidus* (C. L. Koch, 1838). **E.** *Haasea flavescens* (Latzel, 1884). **F.** *Atractosoma meridionale* Fanzago, 1876. **G.** *Cryptops parisi* Brölemann, 1920. **H.** *Henia vesuviana* (Newport, 1845). Photos: J. Spelda.

At the moment our myriapod barcode library includes 320 specimens, 122 species, 56 genera and 24 families of Myriapoda (Fig. 2). All sequences were longer than 500 bp and thus fulfill the requirements for barcoding. The following analysis is based on this dataset (MYFBA), composed of a total of 126 taxa (122 species, three additional subspecies and one subspecies hybrid).

The mean sequence compositions in our sequences are G = 16.32%, C = 21.75%, A = 30.04% and T = 31.87% in Chilopoda, and G = 17.64%, C = 17.67%, A = 26.21% and T = 38.29% in Diplopoda. This shows a pronounced bias towards A and T, which is characteristic of arthropods.

**Figure 3.**
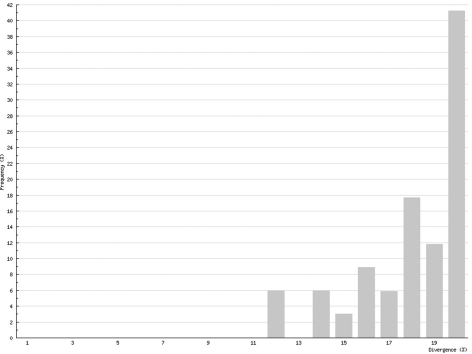
Interspecific COI variability (K2P): distance to nearest neighbour; Chilopoda

**Figure 4.**
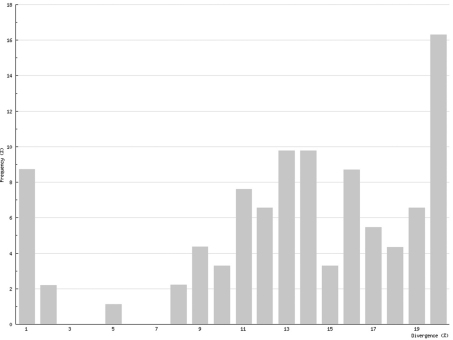
Interspecific COI variability (K2P): distance to nearest neighbour; Diplopoda

In Chilopoda (Fig. 3) the lowest interspecific distance (K2P distance to nearest neighbour) was found between the species *Lithobius borealis* and *Lithobius valesiacus* (11.99%), and the maximum between *Pachymerium ferrugineum* and *Strigamia crassipes* (25.26%). The mean value of the interspecific distance for Chilopoda was 18.30%. Interspecific distances in Diplopoda (Fig. 4) ranged between 0 % in the subspecies of *Craspedosoma rawlinsii* (including the taxa *alemannicum, alsaticum, transsilvanicum* and the hybrid *germanicum* (= *alemannicum X rawlinsii*)) and 33.18 % between the neighbour pair *Polyzonium germanicum* and *Geoglomeris subterranea* which belong to different orders. The mean value of the interspecific distance for Diplopoda was 14.17%.

**Figure 5.**
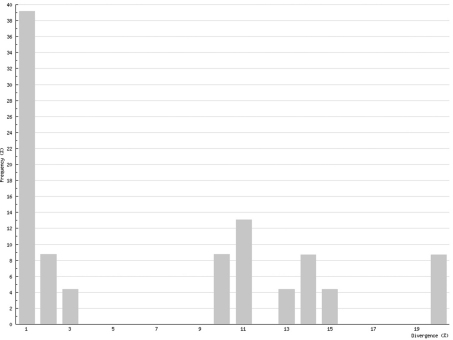
Intraspecific COI variability (K2P): maximum pairwise distances; Chilopoda

**Figure 6.**
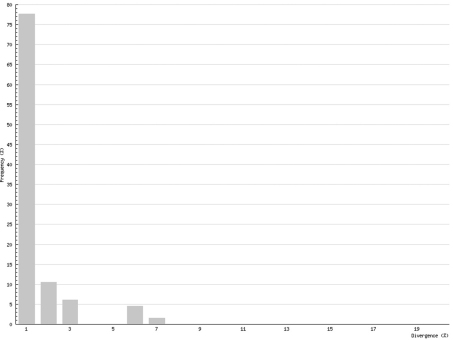
Intraspecific COI variability (K2P): maximum pairwise distances; Diplopoda

Intraspecific distances in Chilopoda (K2P maximum pairwise distance) ranged between 0%, for five species, and 21.55% for *Lithobius microps*, with a mean value for all studied chilopod species of 6.73% (Fig. 5). In Diplopoda, 0% was found for 19 species, and the maximum was 6.61 in *Glomeridella bitaeniata*, with a mean value of 0.82% for all studied diplopods (Fig. 6).

### Neighbour-joining trees

**Figure 7.**
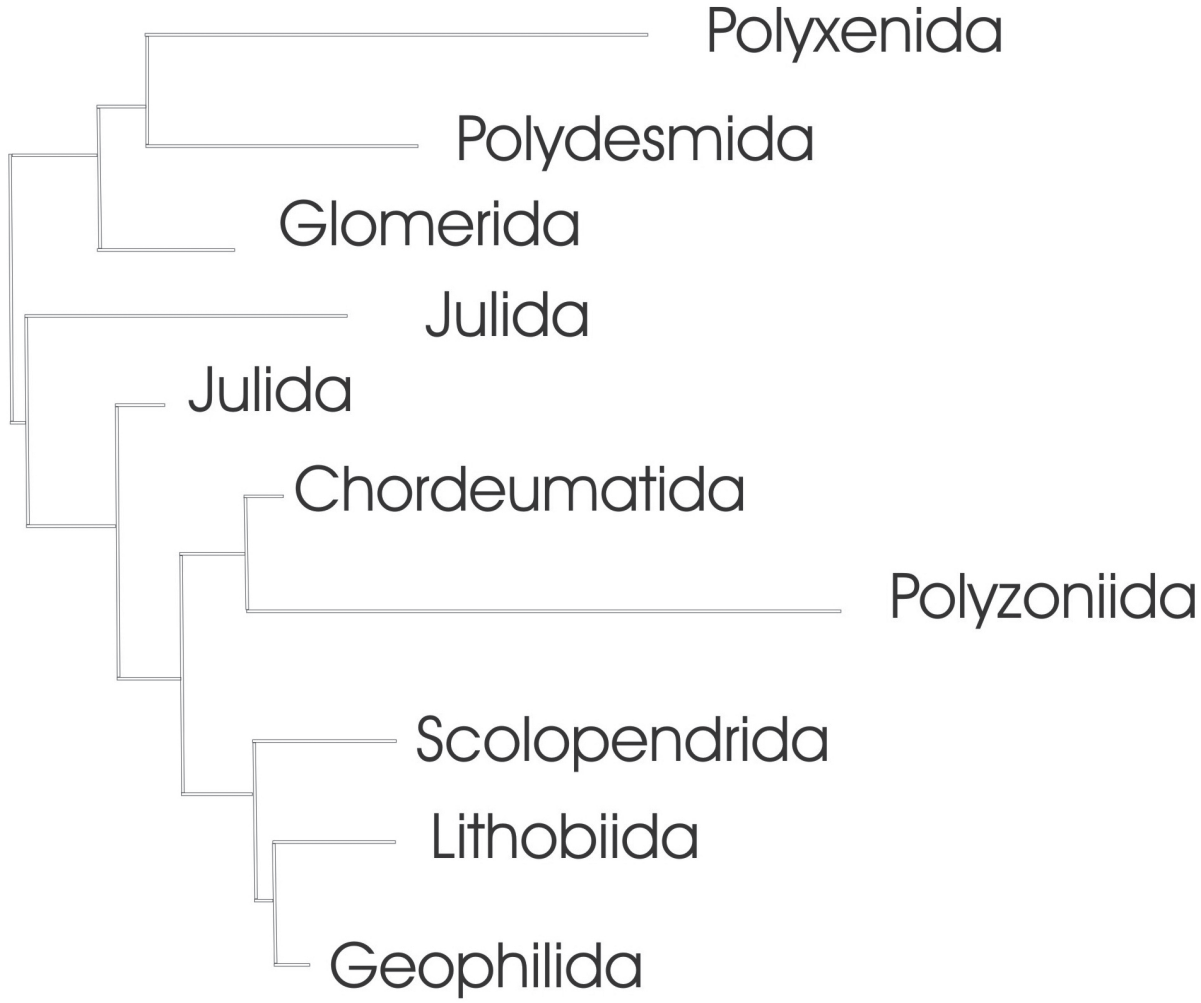
Complete neighbour-joining tree of COI sequence divergences (K2P model) of studied myriapod orders; barcoded terminal taxa and clades above their basal nodes omitted. This tree serves for orientation in the detailed trees given in Figs 8–11.

**Figure 8.**
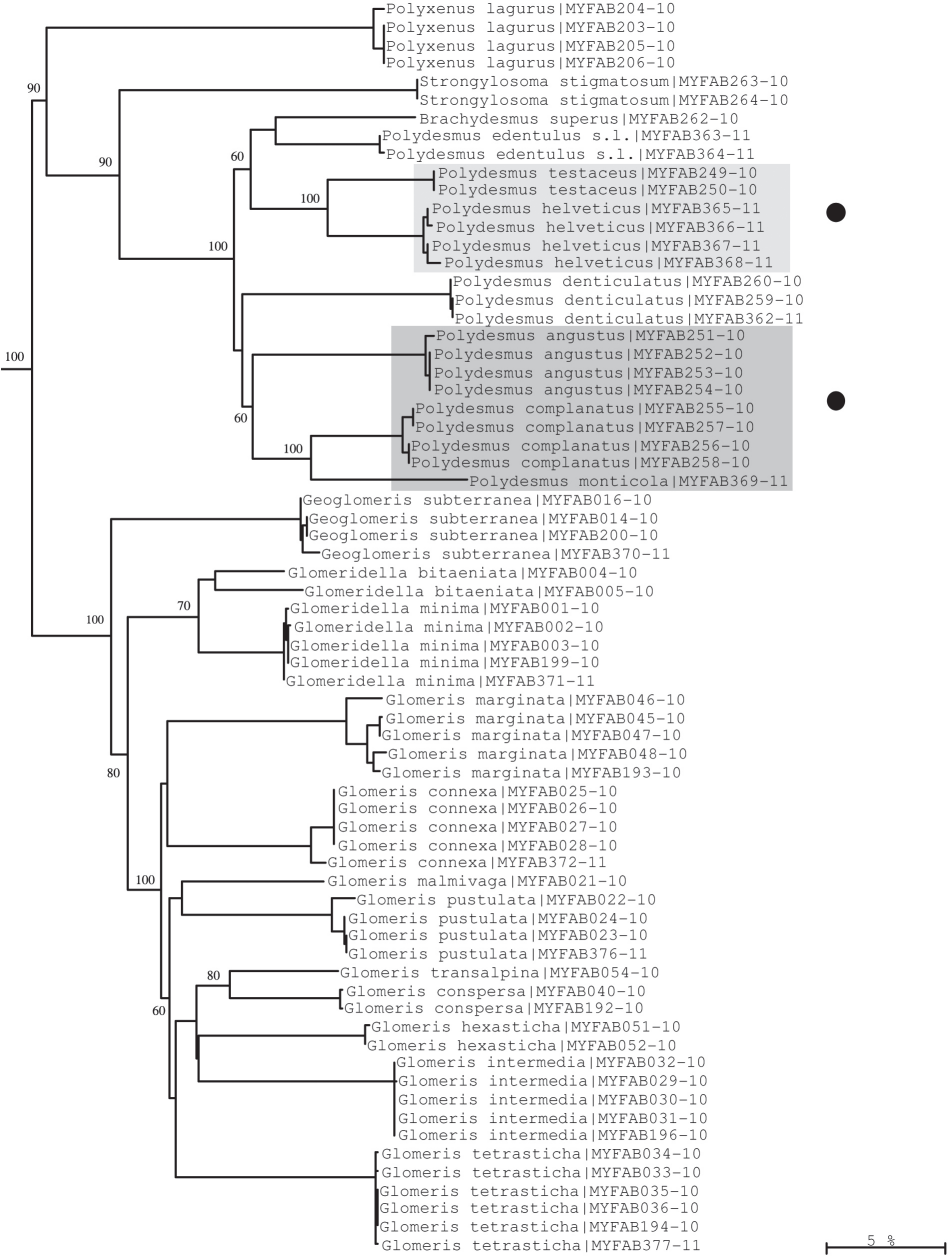
Neighbour-joining tree of COI sequence divergences (K2P model) of studied Polyxenida, Polydesmida and Glomerida. Solid circles: examples of excellent resolution of very close species of the genus *Polydesmus*. Numbers above and below branches show bootstrap values of NJ analysis, branch length indicates sequence divergence in %.

**Figure 9.**
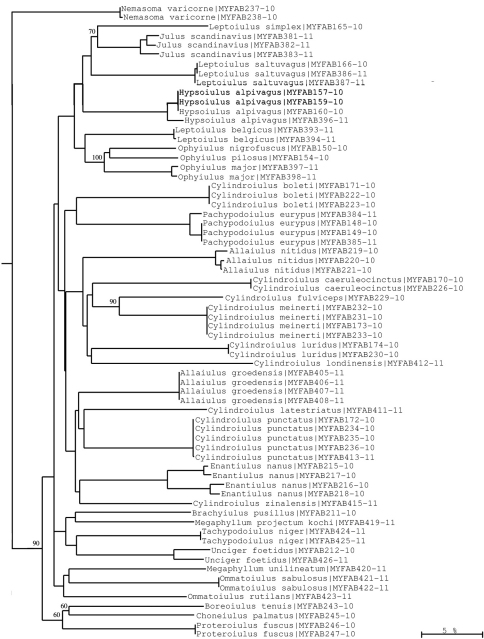
Neighbour-joining tree of COI sequence divergences (K2P model) of studied Julida. Note well-supported COI groups for each species allowing for sequence-based species identification. Numbers above and below branches show bootstrap values of NJ analysis, branch length indicates sequence divergence in %.

**Figure 10.**
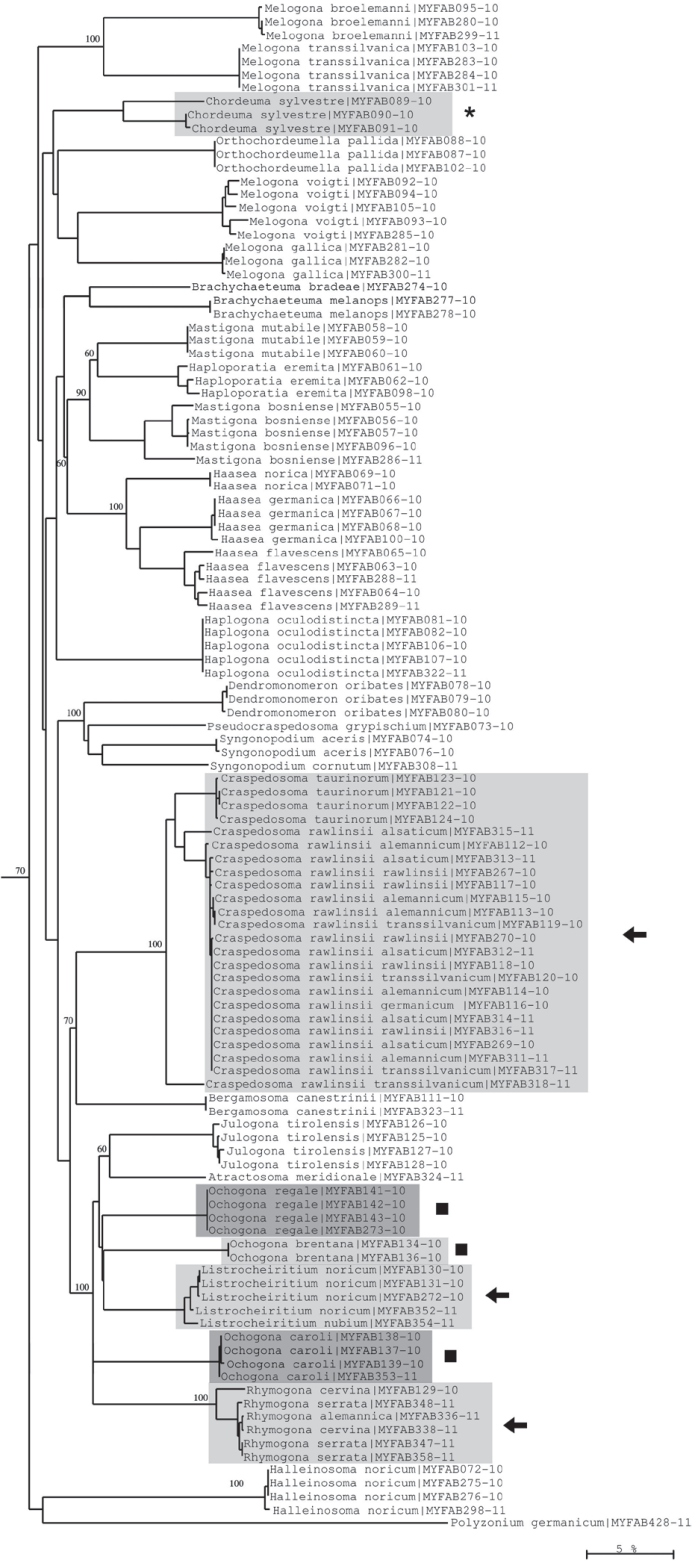
Neighbour-joining tree of COI sequence divergences (K2P model) of studied Chordeumatida. Asterisk: deep barcoding divergence in *Chordeuma silvestre*; solid squares: polyphyly of genus *Ochogona*; arrows: low sequence divergences in the genera *Craspedosoma*, *Listrocheiritium* and *Rhymogona*. Numbers above and below branches show bootstrap values of NJ analysis, branch length indicates sequence divergence in %.

**Figure 11.**
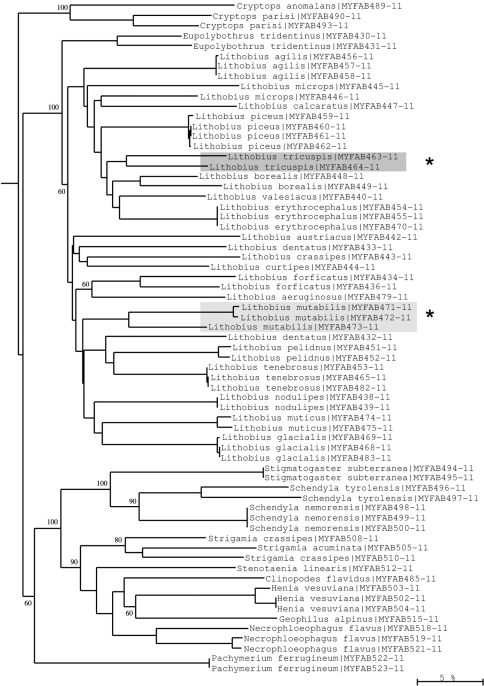
Neighbour-joining tree of COI sequence divergences (K2P model) of studied Chilopoda. Asterisks: Deep divergences within *Lithobius tricuspis* and *Lithobius mutabilis* suggesting cryptic speciation. Numbers above and below branches show bootstrap values of neighbour-joining analysis, branch length indicates sequence divergence in %.

Analysis of our data resulted in the Neighbour-joining (NJ) trees shown in Figs 7–11. Especially in Diplopoda-Helminthomorpha, where species delimitation is comparatively easy due to the diversity of their species-specific secondary copulatory apparatus (gonopods), the results of classical (morphological) taxonomy correspond perfectly with the COI lineages in most cases of our dataset. In the following, we give examples to show how fruitful the combination of barcoding and classical taxonomy can be in myriapod research.

Though the mitochondrial COI gene is generally not seen as adequate for resolving relationships at taxonomic levels higher than species or genus, all barcoded myriapod orders (Polyxenida, Polydesmida, Glomerida, Chordeumatida, Polyzoniida, Scolopendrida, Lithobiida and Geophilida) form single COI clades, except for the Julida, which form two clades (Figs 7, 9). The latter is not too puzzling, however, as according to Enghoff (1981) one of these two clades is formed by the species *Nemasoma varicorne*, which belongs to the only distantly related superfamily Nemasomatoidea. Moreover, several chordeumatidan families are also well supported by the barcodes, i.e. Mastigophorophyllidae, Haaseidae and Craspedosomatidae (Fig. 10).

Most of the studied species appear as distinct COI clades. Barcoded species can overlap for two reasons. First, speciation may have been very recent, e.g. during Pleistocene glacial episodes, as is the case for the diplopod genera *Craspedosoma*, *Rhymogona* and *Listrocheiritium* (Spelda 1996). In these genera genetic introgression is thought to occur commonly. For that reason the subtaxa are treated as subspecies (see Hauser 2004 for *Craspedosoma*, and Scholl and Pedroli-Christen 1996 for *Rhymogona*) or as (semi)species when their separation has been confirmed (Spelda 2006 for *Rhymogona*). The second reason for overlapping barcode groups originates from extraordinarily high intraspecific variation (over 5% divergence) in several nominal chilopod species. Unfortunately, the chilopod samples in our dataset include a comparatively high number of singletons and doubletons, which makes it difficult to decide whether we face cryptic species or genuinely high intraspecific variation. For example, the genus *Strigamia*, especially the *Strigamia crassipes* group, was previously split into many more species than today (Verhoeff 1935), a solution that might be justified in the light of our barcoding results.

At the species and genus levels, we found examples of both well and weakly supported species. For example, *Polydesmus testaceus* and *Polydesmus helveticus* (both often regarded as belonging to a separate genus *Propolydesmus*; see Enghoff and Golovatch 2003), and *Polydesmus angustus*, *Polydesmus illyricus* and *Polydesmus monticola* (*Polydesmus* s. str.), respectively, both form closely related species groups of highly similar morphology that show interspecific COI differences of more than 5%, and hence can be identified unequivocally using DNA barcodes (Fig. 8). It is also interesting to see that two other ‘true’ *Polydesmus* species, *Polydesmus denticulatus* and *Polydesmus edentulus*, are quite distant from both species groups, which implies that the genus *Polydesmus* could be split further.

Conversely, very low interspecific variation is found, e.g., in the chordeumatidan genera *Craspedosoma*, *Listrocheiritium* and *Rhymogona* (Fig. 10). In particular it was not possible to resolve the very closely allied species/subspecies complex within the *Craspedosoma rawlinsii* –group, a result that may reflect ongoing introgression and hybridization. The members of this group even exhibit nearest neighbour distance values of zero, indicating that the COI barcoding method is not suitable for separating its subtaxa.

Moreover, examples of high intraspecific variation can be found in several *Lithobius* species (*Lithobius forficatus*, *Lithobius mutabilis*, *Lithobius tricuspis*) (Fig. 11), and in *Chordeuma sylvestre* (Fig. 10). These deep barcoding divergences could represent more than just high variation and might indicate that cryptic species, previously undetected using the classical morphological approach, are present among our samples. However, the revalidation of the species *Lithobius glacialis* by Pilz et al. (2008) is clearly supported by our barcoding results. This species is distinctly separate from the lowland clade of *Lithobius mutabilis* (a clade that might contain a cryptic species, as stated above), but shows only low intraspecific variation, even though the investigated material originates from very distant mountain areas (Wetterstein Mts Bavaria, and Dachstein Mts Austria). Surprisingly deep divergences are also found within the chordeumatidan genus *Ochogona*, suggesting that this genus is paraphyletic (Fig. 10).

## Discussion

Despite the success of COI barcoding in so many species of centipedes and millipedes it has to be admitted that there are still technical problems with this method that make the success of the barcoding process for any single sample unpredictable. For reasons of cost efficiency the CCDB presently uses only one set of standard primers that are probably not optimal for all groups of centipedes and millipedes. For example, we have failed so far to get any sequences in the genera *Trachysphaera* and *Mycogona*, and have obtained only single chance results in the *Glomeris undulata* and the *Leptoiulus simplex* species groups. The genera *Ommatoiulus*, *Unciger* and *Tachypodoiulus* also seemed to be difficult, as we have obtained only a few barcodes for each of these taxa. To get optimal results special primers would have to be designed. But it is not only the primer design but also the protocol that influences the results. This might explain why some species yielded a barcode in one analytical plate but not in another. Contamination by chemicals (defense secretions in millipedes) might be another cause of unpredictable failures.

Although COI barcoding has provided an excellent tool for the identification of all life stages in several species, there are some problems with this gene locus as it is of mitochondrial origin. This means that it only shows maternal inheritance; therefore different maternal lines might mock cryptic species. This mainly affects the Chilopoda, which show a much higher genetic variability than the Diplopoda. While the histogram of intraspecific distances of the Diplopoda (Fig. 6) resembles that found in insects (e.g., Lepidoptera – Geometridae: Hausmann et al. 2011a), the histogram of the Chilopoda (Fig. 5) implies several undiscovered lineages, either of cryptic species or of long separated haplotypes.

Recent speciations of glacial or postglacial origin with ongoing hybridization and introgression are impossible to resolve using barcodes, as apparently shown by the genera *Craspedosoma*, *Rhymogona* and *Listrocheiritium*. In such cases other genes, especially of nuclear origin, should be used for evolutionary analysis in addition to COI.

Our results show that DNA barcoding can be a highly effective tool for the identification of Chilopoda and Diplopoda, provided that the right primers are designed and the right protocol is used. Before it can be better used, a reference barcode library is needed, the genetic variation must be known, and a close partnership between researchers with taxonomic expertise and those with a background in molecular analysis should be established for the interpretation of the results.
